# Nonacog Alfa for Prophylaxis and Treatment of Bleeding Episodes in Previously Treated Patients with Moderately Severe or Severe Hemophilia B in India

**DOI:** 10.1007/s12288-022-01588-0

**Published:** 2022-11-27

**Authors:** Nirmalkumar Choraria, Savita Rangarajan, M. Joseph John, Shashikant Apte, Pritam Gupta, Seema Pai, Rohit Chand, Shyam Parvatini, G. S. H. Ramakanth, Jeremy Rupon, Amit Chhabra, Hitesh Bhaskarrao Muley, Damien Simoneau

**Affiliations:** 1 Nirmal Hospital Pvt. Ltd, Surat, Gujarat India; 2 K.J. Somaiya Hospital & Research Center, Mumbai, India; 3 University Hospital Southampton NHS Foundation Trust, Southampton, UK; 4 Christian Medical College and Hospital, Ludhiana, India; 5 Pune’s Sahyadri Hospital, Pune, Maharashtra India; 6 Pfizer Healthcare India Pvt. Ltd, Chennai, India; 7 Pfizer Products India Pvt. Ltd, Mumbai, India; 8 Pfizer Inc, Collegeville, PA USA; 9 Pfizer Inc, New York, NY USA; 10 Pfizer IO, Paris, France

**Keywords:** Hemophilia B, Blood coagulation, Factor IX, Recombinant proteins, Treatment outcome

## Abstract

**Purpose:**

Hemophilia B is an X-linked congenital bleeding disorder caused by a deficiency of coagulation factor IX (FIX) clotting activity. This study evaluated safety and efficacy of nonacog alfa, a recombinant human blood coagulation FIX replacement product, in males aged 12–65 years with hemophilia B (FIX activity ≤ 2%) with or without inhibitors in India.

**Methods:**

In this multicenter, open-label, post-approval phase 4 study, participants were treated for up to 8 weeks, with up to a 4-week screening period and a subsequent post-treatment 28-day safety observation period. Intravenous nonacog alfa 40 IU/kg (range 13–78 IU/kg) was administered at intervals of 3–4 days, in accordance with the approved local product document.

**Results:**

A total of 25 participants were enrolled and completed the study. No participants developed FIX inhibitors during the study, experienced treatment-related adverse events (AEs) or serious AEs, or developed a thrombotic event and/or hypersensitivity reaction. No participants experienced bleeding events requiring on-demand treatment with nonacog alfa. Seventeen bleeding episodes (16 spontaneous and 1 traumatic) were reported in 10 participants; all occurred post treatment, with the exception of a minor gum-bleeding event, and were managed without treatment. The mean (SD) annualized total factor consumption (TFC) per patient was 224,582 (75,527) IU; the mean (SD) annualized TFC by weight per patient was 3639 (573) IU/kg.

**Conclusion:**

Nonacog alfa was safe and effective for the prevention of hemorrhagic episodes in Indian males with congenital, severe hemophilia B. No participants developed FIX inhibitors, and no new safety signals were reported.

## Introduction

Hemophilia B is an X-linked congenital bleeding disorder caused by a deficiency of coagulation factor IX (FIX) clotting activity, which can manifest as bruising, spontaneous bleeding events, or excessive bleeding after an injury or surgery, particularly into muscles, joints, and soft tissues [1, 2]. Hemophilia B accounts for up to 20% of all cases of hemophilia [1].

According to a World Federation of Hemophilia (WFH) global survey, in 2020, India had 3150 individuals with hemophilia B (incidence: 0.23 per 100,000), second only to the United States (n = 4093; incidence: 1.25 per 100,000) [3]. However, the actual incidence rate in India is probably higher than the rate reported by the WFH. In India, a substantial proportion of individuals with hemophilia remain undiagnosed, with many cases unregistered, suggesting that these rates are likely underestimated [4]. By comparison, the proportion of individuals with hemophilia B in 2020 was approximately 10-fold lower in other Asian countries, including Indonesia (n = 347; incidence of 0.13 per 100,000), the Philippines (n = 201; 0.19 per 100,000), Thailand (n = 206; 0.30 per 100,000), and Malaysia (n = 177; 0.55 per 100,000) [3]. Given the poor availability of diagnostic facilities, a diagnosis of hemophilia can be missed leading to a low rate of diagnosis and/or late diagnosis so by the time of diagnosis, Indians with hemophilia may have accrued joint damage due to the lack of treatment in the absence of a diagnosis [4].

Available treatment options for individuals with hemophilia B include plasma-derived or recombinant clotting factor concentrate, administered on demand or as prophylaxis treatment [1, 5]. Although current plasma-derived products have a good safety profile, concerns remain regarding the potential for transmission of infectious diseases; this has led to a transition toward greater use of recombinant products [1, 5]. However, the requirement for multiple infusions of factor replacement in some individuals can limit adherence and, thus, the efficacy of these therapies [5].

The use of both standard and extended half-life FIX replacement products is much lower per capita in India than in other parts of Asia and the rest of the world [3]; recent 2020 WFH estimates for mean per capita FIX use in 2019 were 0.015 IU/total population in India compared with estimates ranging from 0.0004 IU/total population in Indonesia to 0.400 IU/total population in Singapore, 1.575 in the United States, and 1.138 in the United Kingdom [3]. Economic considerations drive treatment decisions in Indians with hemophilia, with few individuals having access to factor replacement therapy and subsequent reliance on adjunct therapies such as tranexamic acid and products such as fresh frozen plasma and cryoprecipitate [4]. Development of inhibitors has also been reported in Indian patients with hemophilia, including those with hemophilia B, with prevalence rates of up to 13% [4]. Gaining an understanding of inhibitor development in these patients is hindered by the inconsistent administration of factor replacement therapy and frequent switching of products.

Nonacog alfa, a standard half-life recombinant human blood coagulation FIX replacement product, is approved in India for the treatment and prophylaxis of bleeding events in patients with hemophilia B [6, 7]. Although extended half-life replacement FIX products are available, access to these products is limited in India so the need for standard half-life products recombinant or plasma-derived products is still high. Therefore, this post-approval phase 4 study evaluated the safety and efficacy of nonacog alfa in males with moderately severe or severe hemophilia B in India.

## Methods

### Study Design

This single-country, multicenter, open-label, single-arm interventional study was conducted between February 10, 2020, and September 24, 2020. Participants were treated for up to 8 weeks, with up to a 4-week screening period and a subsequent post-treatment 28-day safety observation period. Participants continued in the study until at least 16 exposure days (EDs) or a period of up to 8 weeks on nonacog alfa (whichever occurred first). Intravenous nonacog alfa 40 IU/kg (range 13–78 IU/kg) was administered at intervals of 3–4 days, in accordance with the approved local product document (LPD). For on-demand treatment, the amount and frequency of administration were individually tailored by the investigators according to their own judgement.

### Participants

Males aged 12 through 65 years with congenital moderately severe to severe hemophilia B (FIX activity ≤ 2%) and a documented history of ≥ 50 EDs to FIX-containing products were included.

Participants were excluded if they had a history of FIX inhibitors or positive inhibitor testing (≥ 0.6 Bethesda unit [BU]/mL) during screening, clinical signs or symptoms of a decreased response to FIX, or any bleeding disorder other than hemophilia B, were immunocompromised with HIV, had planned surgery within 6 months of the study initiation, or participated in other studies involving other investigational drugs within 30 days of study initiation.

### End Points

The safety end points included FIX inhibitor development (primary end point) and the incidence of serious adverse events (SAEs), including thrombotic events, hypersensitivity reactions, and treatment-emergent adverse events (TEAEs), classified according to the Medical Dictionary of Regulatory Activities (MedDRA) v23.1; TEAEs were monitored from the administration of first dose of study drug until treatment end date plus 28 days. Efficacy end points included the annualized bleeding rate (ABR) during prophylaxis, calculated based on bleeding events that required on-demand infusion with nonacog alfa while receiving nonacog alfa as prophylaxis, annualized total factor consumption (TFC, IU/kg body weight), and the number of nonacog alfa infusions used to treat each new bleeding episode.

### Statistical Analysis

Participants who received at least 1 dose of nonacog alfa were included in the safety analyses; those who received at least 1 dose of nonacog alfa and had evaluable data for the corresponding end point were included in the efficacy end point analyses.

Data were summarized using descriptive statistics. The primary endpoint for this study was the proportion of participants for which FIX inhibitors were detected at visit 4. Because of the size and nature of this study, a 2-sided 90% confidence interval (CI) was chosen for the corresponding proportion which was computed using the exact method. For annualized TFC by weight, dose by weight was summed per subject then annualized, i.e. (total units by weight / treatment interval duration) × 365.25, then used for descriptive statistics calculation. Post hoc analyses computed the proportion of participants who had no treated bleeding events during the prophylaxis period; 95% CIs were calculated applying the exact method.

## Results

### Study Population

Of the 30 screened participants, 25 were enrolled and completed the study. Baseline characteristics are described in Table 1. All 25 enrolled participants were males of Indian Asian origin, with a mean (standard deviation [SD]) age of 28.4 (11.0) years. In total, 12 (48%) participants had a family history of hemophilia. Only 1 (4%) participant had a family history of inhibitors to FIX products, and none had a history of allergy to FIX products. All 25 (100%) participants were classified as having severe disease, based on the investigator’s assessment of clinical bleeding symptoms.

**Table 1:** Demographics and baseline characteristics (safety analysis population)

Parameter	Results (N = 25)
Age	
Mean (SD), years	28.4 (11.0)
Median (range), years	25 (12–51)
Indian race, n (%)	25 (100)
Body mass index, median (range), kg/m^2^	21.5 (14.8–35.2)
Factor IX activity, median (range), %	0.499 (0.5–2.50)
Mean (SD) laboratory values	
ALT (U/L)	0.585 (0.5031)
AST (U/L)	0.407 (0.1621)
Bilirubin (µmol/L)	11.04 (5.731)
ALP (U/L)	1.834 (0.7517)

ALP, alkaline phosphatase; ALT, alanine aminotransferase; AST, aspartate aminotransferase; SD, standard deviation

A total of 24 (96%) participants had identified target joints at screening: the knees, elbows, and shoulders were the most prevalent target joints. Nine (36%) participants had hemophilic arthropathy affecting the knees, elbows, and ankles. The median (min–max) number of prior FIX EDs was 77.0 (50–500) days (mean [SD] 112.6 [108.9] days ). All participants received 16 prophylactic infusions: mean (SD) dose by weight per infusion was 37.6 (4.3) IU/kg (median [min–max]: 39.29 IU/kg [25.0–43.6]).

### Safety

No participants developed FIX inhibitors (≥ 0.6 BU/mL) during the course of the study, as confirmed by central laboratory testing. A 2-sided 90% CI using the exact method for the proportion of participants for which FIX inhibitors were detected at visit 4 was computed; the upper and lower bounds were 0.00% and 0.11%, respectively, confirming that the likelihood of a participant developing FIX inhibitors is < 0.1%. TEAEs were reported in 3 (12%) participants; 2 cases were mild (pyrexia, n = 1; cough, n = 1), and 1 was of moderate severity (dental caries) (Table 2). The safety event of pyrexia was determined by the investigator to be mild in severity and was not considered related to the study intervention. TEAEs were not considered by investigators to be related to nonacog alfa or to hemophilia B. No TEAE-related dose reductions or discontinuations attributed to SAEs were reported. No patient developed a thrombotic event or hypersensitivity reaction.

**Table 2:** Treatment-emergent adverse events

Parameter	Results (N = 25)
Participants with AEs, n (%)	3 (12)
Mild	2 (8)
Moderate	1 (4)
Treatment-related AE, n (%)	0 (0)
SAE, n (%)	0 (0)
Withdrawal because of AEs, n (%)	0 (0)
AE^a^, n (%)	
Cough	1 (2)
Dental caries	1 (2)
Pyrexia	1 (2)

AE, adverse event; SAE, serious adverse event

^a^If the same patient in a given treatment had more than 1 occurrence in the same preferred term event category, only the most severe occurrence was counted; participants were counted once per treatment per event

### Clinical Outcomes

During the treatment phase of the study, no participants experienced bleeding events requiring on-demand treatment with nonacog alfa (ABR, 0). In total, 17 bleeding episodes (16 spontaneous and 1 traumatic) were reported in 10 participants; all occurred post-treatment (i.e. after ≥ 16 EDs or 8 weeks [whichever occurred first]), with the exception of a minor gum bleed that occurred on the 37th day from start of study drug (i.e., during the 28-day safety observation period post the study treatment phase) and was managed without treatment. None of the bleeding events that occurred post-treatment necessitating FIX infusions were treated with nonacog alfa; 15 of these bleeding episodes were treated with other FIX products, and 2 were minor bleeding events that resolved without treatment. The mean (SD) annualized TFC per patient was 224,582 (75,527) IU; the mean (SD) annualized TFC by weight per patient was 3639 (573) IU/kg.

## Discussion

Despite the high proportion of individuals with hemophilia B in India, there is a paradoxical low per capita use of FIX treatment, which may indicate an unmet need for effective prophylactic regimens in this patient population [3, 8]. The Hemophilia Foundation of India acknowledges the importance of regular prophylaxis or replacement therapy in individuals with hemophilia but also notes that this may be a challenge to implement as part of routine clinical care in India [8]. Effective recombinant therapies with a lower risk of inhibitor development are needed.

This post-approval phase 4 study supports the safety and efficacy of nonacog alfa when administered as prophylaxis in Indian males with moderately severe to severe hemophilia B with regard to hemostatic control and prevention of bleeding events when used in accordance with the approved LPD. No patient developed FIX inhibitors during the study, as confirmed by central laboratory analysis.

The observed safety profile of nonacog alfa in this study was consistent with previous studies [9, 10]. Nonacog alfa was well tolerated, with only 3 (12%) participants experiencing an AE. All of the reported AEs were mild to moderate in nature, and none were considered treatment- or hemophilia-related. No bleeding episodes (ABR = 0) required an on-demand infusion of nonacog alfa during the treatment phase of the study. Although 1 participant had a family history of inhibitors, no participant developed inhibitors during the course of the study. This is a particularly important consideration given the inherent costs associated with detection and treatment of inhibitors, particularly in lower-income health care settings [4, 8].

Recombinant prophylaxis was similarly well tolerated in a phase 3 study of trenonacog alfa in individuals with previously treated hemophilia B, which included participants from India [11]. The most common AE in this cohort was headache (2.6%), and no participant developed inhibitors during the study. Although 21% of the participants developed transient noninhibitory binding FIX antibodies following screening, they were not considered clinically relevant. Routine prophylaxis in this study (n = 61; median duration 16.2 months) was associated with a median ABR of 1.52, and 31% (19/61) of participants who received prophylaxis had no bleeding episodes reported [11].

A potential limitation of this study was its relatively short duration (8 weeks), which did not allow for longer-term data collection to assess the efficacy of nonacog alfa for bleeding prevention. Nevertheless, these findings provide valuable insight on the efficacy of nonacog alfa in Indian patients with hemophilia B and contribute to the substantial body of experience with nonacog alfa in the real-world setting [10, 12].

In conclusion, nonacog alfa was safe and effective in preventing hemorrhagic episodes in Indian males with hemophilia B when used according to the LPD. No patient developed FIX inhibitors during the study, and there were no new safety signals.

## Data Availability

Upon request, and subject to review, Pfizer will provide the data that support the findings of this study. Subject to certain criteria, conditions, and exceptions, Pfizer may also provide access to the related individual de-identified participant data. See https://www.pfizer.com/science/clinical-trials/trial-data-and-results for more information.
